# Clinical outcomes following responsive neurostimulation implantation: a single center experience

**DOI:** 10.3389/fneur.2023.1240380

**Published:** 2023-09-22

**Authors:** Micaela R. Owens, Michael Sather, Tiffany L. Fisher

**Affiliations:** Department of Neurology, Penn State Health Milton S. Hershey Medical Center, Hershey, PA, United States

**Keywords:** epilepsy, RNS, responsive neurostimulation, StereoEEG, neuromodulation

## Abstract

**Background:**

Responsive neurostimulation (RNS) is an implantable device for persons with medically refractory focal-onset epilepsy. We report a single-center experience for RNS outcomes with special focus on stereoelectroencephalography (sEEG) for seizure onset localization.

**Methods:**

We performed retrospective review of patients with drug resistant focal epilepsy implanted with the RNS System for a minimum of six months between July 2014 and July 2019. Records were reviewed for demographic data, epilepsy duration, seizure frequency, number of prior antiepileptic drugs (AEDs), number of AEDs at RNS System implantation, prior epilepsy surgery or device use, previous seizure localization with sEEG, and RNS system information. Clinical response was defined as a 50% reduction in seizures. Differing response rates were calculated using Fisher Exact test.

**Results:**

30 patients met inclusion criteria. Seventeen (57%) underwent previous sEEG. Average clinical follow up was 3.0 years. Overall response rate was 70%. Median seizure reduction was 74.5%. Response rate was 82.3% for patients with sEEG compared to 53.8% without (*p* = 0.08); 37.5% for prior epilepsy surgery compared to 81.8% without (*p* = 0.02); 70% for mesial temporal onset; 50% for previous vagal nerve stimulator compared to 77.3% without (*p* = 0.13).

**Conclusion:**

Our response rates match or surpass outcome metrics of previous studies. Although limited by small study size, subpopulation analyses show positive response rates in patients with previous sEEG versus no sEEG and in temporal versus extratemporal pathology. Additional research is needed to evaluate efficacy of RNS in patients with previous epilepsy surgery, and utility of sEEG in this population.

## 1. Introduction

More than 30% of persons with epilepsy develop medically refractory disease, meaning they will continue to have seizures despite adequate trials of two appropriate anti-epileptic drugs (AEDs) (1). Responsive neurostimulation (RNS) is readily available to patients with medically refractory focal-onset epilepsy. Real-world RNS efficacy has outpaced the original treatment responses seen in clinical trials, with possible explanations including improved detection and stimulation programming with increased clinical experience (2). Use of stereotactic electroencephalography increases the accuracy of seizure onset localization and thus may improve treatment outcomes with RNS. The goal of the current paper is to analyze treatment responses at a center experienced in stereoelectroencephalography (sEEG).

## 2. Background

The RNS (NeuroPace) system is approved for the treatment of medically refractory focal-onset epilepsy. Patients are typically considered for RNS if seizures are localized to 1 or 2 foci and there are at least three or more disabling seizures per month on average despite AEDs (these may include focal motor, focal onset with impaired awareness, and secondarily generalized tonic–clonic seizures). The system is comprised of a cranially implanted programmable neurostimulator allowing implantation of a maximum of four leads – depth electrodes or subdural strips. However, there is a limitation of two that can be connected for recording and stimulating the patient’s specific seizure focus. The neurostimulator continually senses electrocorticographic activity and delivers stimulation in response to abnormal activity according to parameters specified by the physician. Both seizure detection and stimulation parameters are tailored to the patient and modified over time for optimal seizure control (3).

RNS efficacy was established in the randomized, multicenter, double-blinded, sham-stimulation controlled pivotal trial, with final results of up to 2 years postimplant follow-up data released in 2014 (4). The blinded assessment period lasted 12 weeks, during which time the treatment group experienced a significantly greater reduction in total disabling seizures compared to sham group (41.5% and 9.4% reductions respectively). During the open label period, median percent reduction reached 44% at 1 year, and 53% reduction at 2 years. The overall responder rate, the percentage of patients with at least a 50% reduction in clinical seizure frequency, was 54%. The study was not powered for subgroup analysis but descriptively found no major differences based on mesial temporal lobe onset or changes in anti-seizure medications. Quality of life outcome measures were also favorable. Subsequent real-world experience with RNS has demonstrated median seizure frequency reduction of 67% at 1 year and 75% at 2 years. Responder rate was 66% at 1 year.

The advantages of RNS include safety compared to traditional epilepsy surgery in eloquent cortex, preservation of or even improvement in cognition over time, comparable hemorrhage and infection rates to other intracranial surgeries, overall excellent long-term tolerability (2, 4), and potentially favorable patient perception of invasiveness. As with any new procedural therapy, the primary disadvantage is accessibility.

Traditional indications for intracranial EEG include localization of seizure foci in (a) nonlesional epilepsy, (b) large/deep/multifocal lesions, (c) epileptic zone in proximity to eloquent cortex, and (d) previous failed surgery. SEEG offers less coverage of superficial cortical areas compared to subdural strip- or grid-electrodes; however, it does allow bilateral symmetric implants for better sampling of epileptic networks and precise mapping of deep cortical areas. SEEG is also usually better tolerated and carries a lower rate of clinically significant complications. Use of sEEG has improved study of large, deep, and multifocal lesions including polymicrogyria and heterotopic gray matter, and in surgical planning of suspected bitemporal lobe epilepsy (5). There have been some studies highlighting patterns on sEEG that might predict response to RNS (6), however the role for sEEG in preoperative evaluation for RNS if resection is not intended remains unclear. We hypothesized that precise seizure onset localization using sEEG could guide RNS implantation and improve RNS response rates.

## 3. Methods

### 3.1. Patient selection

We performed retrospective review of patients with drug resistant focal epilepsy who were implanted with the RNS System for a minimum of 6 months between July 2014 and July 2019 at the Pennsylvania State University Hershey Medical Center (PSUHMC), a comprehensive level 4 epilepsy center. Patients required at least six months of outcomes data after implantation to ensure that the “implant effect” from surgery was bypassed (3). All implanted patients with the minimum six months of post-implantation data were included in the analysis. Electronic medical records were reviewed for age, sex, epilepsy duration, seizure frequency, number of prior antiepileptic drugs, number of AEDs at RNS System implantation, prior epilepsy surgery or device use, previous seizure localization with sEEG, and RNS system information including lead type and location. All patients were discussed at an interdisciplinary epilepsy case conference prior to implantation of the RNS System. Guidelines for consideration of RNS at PSUHMC adhere to the selection criteria in the pivotal trial (3, 4), including a minimum of 3 disabling seizures per month. We consider RNS placement in patients with lower-frequency events meeting certain exceptional circumstances: if seizures present with severe injury or status epilepticus and (a) other interventions such as VNS, laser ablation, or resective surgery have already failed or (b) the patient is not a candidate for resection or ablation.

### 3.2. Data collection and analysis

Baseline clinical seizure frequency was the patient-reported number of seizures with impaired awareness with and without secondary generalization prior to RNS System implantation. Focal aware seizures were not counted when determining seizure frequency. Clinical seizure frequency was retrospectively assessed based on documentation by treating providers at each outpatient follow up visit based on seizure diaries and/or self-report from the patient or caregiver. Rates of seizure reduction or increase for each patient were determined and then compiled into response categories of increased seizures, seizure freedom, and seizure reduction quartiles ranging from 0% to 99%. Responder rate of patients with at least a 50% reduction in clinical seizure frequency is taken at last observation.

Statistical tests used for data analysis are indicated in the text. For all comparisons α was set to *p* < 0.05 for statistical significance. Subpopulation analysis of response rates was completed for patients with mesial temporal onset including mesial temporal sclerosis (MTS), sEEG, prior epilepsy surgery, and previous vagus nerve stimulator (VNS).

## 4. Results

Over a five-year period, a total of 31 patients were implanted with the RNS System at PSUHMC and carried a diagnosis of medical refractory localization related epilepsy with disabling seizures. However, there were two pediatric patients (age < 18 years) at the time of implantation for which the device is not approved. Three patients underwent revision of RNS leads due to a single lead break, one of whom was excluded due to less than six months of outcomes data following revision. Specific clinical characteristics for each patient are provided in Table 1. Of the 30 remaining patients, 40% (*n* = 12) were female. Mean age at time of RNS System implantation was 33.4 years ±10.7 (range 14–55). Mean duration of epilepsy was 20.5 ± 9.4 years (range 7–41 years). Patients were taking a mean of 3.0 ± 1.0 (range 1–6) antiepileptic drugs at the time of implantation with a mean of 4.7 ± 1.6 (range 2–9) antiepileptic drugs tried previously. The majority of patients (20, 66.7%) had onset of seizures from the mesial temporal region and 13 patients (43.3%) had two seizure foci. Seventeen of 30 patients (57%) had prior intracranial monitoring for seizure localization with stereotactic EEG (sEEG). There were a small cohort of patients who previously underwent either resection or laser ablation (8, 27%), vagal nerve stimulator placement (8, 27%), or both (2, 6.7%). Grouped characteristics for 30 implanted patients can be found in the Supplementary material.

**Table 1:** Individual patient characteristics.

Case no.	Years with epilepsy	AED no.	Baseline seizure frequency	Prior surgery	Prior VNS	Foci no.	sEEG	RNS location (strip/depth)	Current seizure frequency	Percent reduction
1	18	6	10	Lobectomy	Y	1	N	S – L Post Basal Temporal S – L Inf Lat Temporal	7.1	29.00
2	13	4	13.5	N	N	2	Y	D – Bil Mesial Temporal	4.5	66.67
3	29	3	75	N	N	1	N	D – L Ant Temporal D L Post Temporal	7.8	89.60
4	19	3	8	N	N	2	Y	D – L Mesial Temporal S – R Mid Temporal Gyrus	2.7	66.25
5	10	3	32	N	N	2	Y	D – Bil Mesial Temporal	5	84.38
6	13	2	10	N	N	1	N	D – R Mesial Temporal S – R Inf Occipital	4.25	57.50
7	8	4	3	N	N	2	Y	D – Bil Mesial Temporal	0	100.00
8	7	4	30	N	N	1	Y	D – R Ant Inf Parietal S – R Inf Parietal	1	96.67
9	24	3	6	N	Y	1	Y	S – L Mid Frontal Gyrus	3.7	38.33
10	10	4	80	N	Y	1	Y	S – L Frontal (Motor) L Sup Temporal Gyrus	10.75	86.56
11	30	5	70	N	Y	2	Y	D – Bil Mesial Temporal	6.5	90.71
12	26	4	75	Lobectomy	N	1	N	D – L Mesial Temporal	14	81.33
13	41	3	95	N	Y	2	N	D – Bil Mesial Temporal	1.5	98.42
14	34	3	8	Lobectomy	N	1	Y	D – R Post Orbitofrontal D – R Frontal (Premotor)	2.3	71.25
15	8	4	28	N	N	2	Y	S – Bil Mid Frontal Gyrus	3.8	86.43
16	25	3	2.5	N	N	1	Y	S – L Orbitofrontal S – L Frontal Operculum	0	100.00
17	36	3	5.5	N	N	2	Y	D – L Mid Frontal Gyrus D – L Mesial Temporal	0.5	90.91
18	25	3	45	N	N	1	N	D – L Mesial Temporal	5	88.89
19	10	2	0.75	Ablation	N	1	N	D – L Mesial Temporal	6	−700.00
20	20	4	5	Lobectomy	Y	2	N	D – L Mesial Temporal	1.3	74.00
21	34	2	1.3	Lobectomy	N	1	N	D – R Mesial Temporal	0.7	46.15
22	15	2	90	N	Y	1	Y	D – L Mesial Temporal S – L Lat Temporal	0.71	99.21
23	16	1	1.5	N	N	1	N	D – R Mesial Temporal	12.5	−733.33
24	14	2	6	N	N	2	N	D – Bil Mesial temporal	1.5	75.00
25	17	2	10.5	N	N	1	Y	D – R Mesial Temporal S – R Temporal	3.7	64.76
26	28	2	4	N	N	2	Y	D – L Insulotemporal D – R Mesial Temporal	0.5	87.50
27	8	2	20	N	N	2	N	D – Bil Mesial Temporal	15	25.00
28	27	3	12	N	Y	2	Y	D – R Post Cing Gyrus S – R Frontal (Motor)	15	−25.00
29	24	3	0.5	Lobectomy	N	1	N	D – R Mesial Temporal	4	−700.00
30	27	1	9.2	Lobectomy	N	1	Y	D – L Post Sup Temporal Gyrus D – R Mesial Temporal	7.8	15.22

A total of 60 leads were implanted of which 16 were strips and the remaining 44 were depth leads accounting for 19 dual depth systems, 5 dual strip systems, and 6 combination systems with both depth and strip leads. The majority of leads were implanted in the mesial temporal lobe (*n* = 33) with other locations consisting of the frontal (*n* = 11), parietal (*n* = 4), lateral temporal (*n* = 10), occipital (*n* = 1), and insular regions (*n* = 1).

### 4.1. Seizure reduction

The median baseline seizure frequency prior to implantation of the RNS device was 10 (range 0.5–90) per month. There were 4 patients with seizure frequency of <2 per month and 2 patients with <1 seizures per month. Three of these 4 patients with low baseline seizure frequency were selected for RNS due to continued disabling seizures following lobectomy. One patient (number 23 in the table) had a seizure frequency of up to 4 per month at the time of epilepsy surgery conference, and seizure frequency declined in the interim before RNS System implantation as medications were adjusted. All patients were followed for a minimum of six months with a mean follow up of 3.0 years and a cumulative of 90 patient implant years.

**Figure 1:**
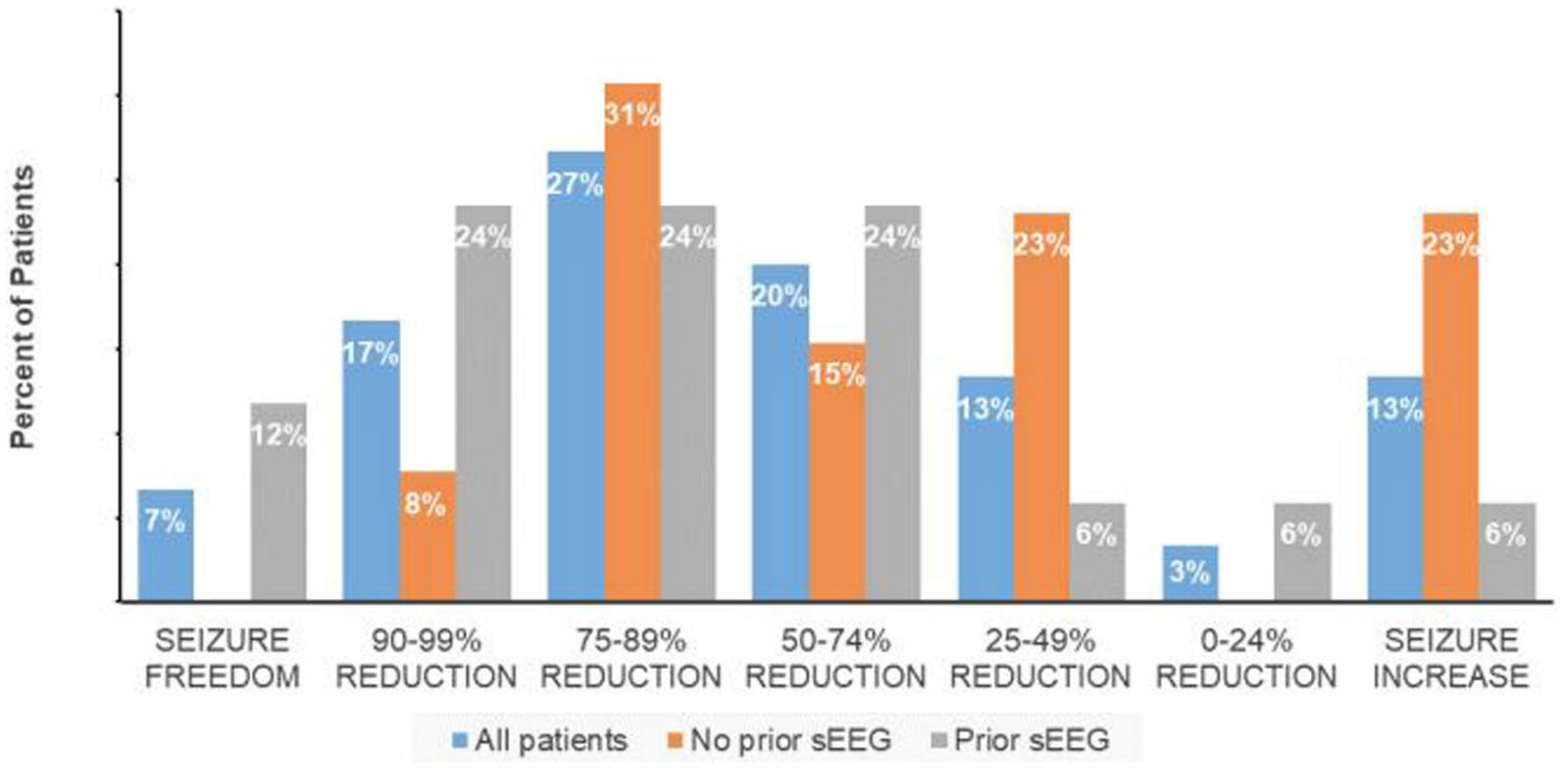
Response quartiles (all implanted vs. sEEG localization).

The responder rate (percent of subjects with at least a 50% reduction in seizure frequency) was 70% with a median reduction of 74.5%, and median seizure frequency of 3.9 (range 0–15) per month. For the group with less than a 50% response rate, there was an equal percentage of patients with increased seizure frequency and a 25%–49% seizure frequency reduction while 3% had less than a 25% reduction in seizures (Figure 1). Non-responders were on average slightly younger (mean 32.5 years) with a similar epilepsy duration (20.8 years).

### 4.2. Subpopulations

The 17 patients who underwent sEEG for seizure localization just prior to RNS implantation have a responder rate of 82.3% in comparison to 53.8% for the patients with no prior invasive monitoring for seizure localization (one-tailed Fisher exact test, *p* = 0.08; Figure 1). The median seizure reduction was 86.4%, and median seizure frequency fell from 10.5 to 3.7. There were four patients (13%) with at least a 95% reduction in seizures and two patients (6.7%) with no seizures for at least six months. One patient (0.6%) experienced an increase in seizure frequency from 12 to 15 per month. Outcomes for specified subpopulations are summarized in Table 2.

**Table 2:** Subpopulation analysis.

Subpopulation	50% responder rate (%)	Median % reduction	Minimum % reduction	Maximum % reduction	*p*-value (responder rate)
Total participants (*n* = 30)	70	74.5	−733.3	100	
sEEG (*n* = 17)	82.3	86.4	−25	100	0.08
Prior surgery (*n* = 8)	37.5	37.5	−700	81.3	**0.02**
Mesial temporal onset (*n* = 20)	70	70.3	−733.3	100	
Radiographic mesial temporal sclerosis (*n* = 13)	61.5	66.3	−733.33	99.2	0.35
Prior VNS (*n* = 8)	50	80.3	−25	99.2	0.13

Statistically significant result is shown in bold.

The eight patients who had a prior history of epilepsy surgery (including 7 lobectomies and 1 ablation) have a responder rate of 37.5%, compared to 81.8% for the patients with no history of prior epilepsy surgery (Fisher exact test, *p* = 0.02). The median seizure reduction was 37.5%, and median seizure frequency fell from 6.5 to 5. Maximum seizure reduction was 81.3%, and two patients with relatively low seizure frequency at baseline (<1 per month) experienced an increase in seizure frequency. Only two patients underwent sEEG prior to implantation in this group. Both experienced a decrease in seizure frequency but in only one patient was the decline >50%.

The 20 patients with mesial temporal localization included 13 patients with imaging findings consistent with MTS, 1 patient with hippocampal cortical dysplasia, and 6 patients with normal imaging. This group had an overall responder rate of 70%, equal to the population as a whole. The 13 patients with MTS have a 61.5% response rate compared to 85.7% response rate in the non-MTS group (Fisher exact test, *p* = 0.23). The median seizure reduction was 70.3% overall, 66.3% in the MTS group, and 81.3% in the non-MTS group. Median seizure frequency fell from 9.6 overall, 9.2 in the MTS group, and 10.5 in the non-MTS group to 4.1, 4.3, and 3.7, respectively. There were two patients in the MTS group (16.7%) and one in the non-MTS group (12.5%) with at least a 95% reduction in seizures, and one patient (12.5%) in the non-MTS group with no seizures for at least six months. Three MTS patients with relatively low seizure frequencies at baseline experienced an increase in seizure frequency. Nine patients underwent sEEG prior to implantation, with an 88.9% response rate compared to 54.5% response rate without prior sEEG (Fisher exact test, *p* = 0.11), with similar rates between the MTS and non-MTS groups.

The eight patients with previous vagal nerve stimulator have a responder rate of 50%, compared to 77.3% for the patients without prior VNS (Fisher exact test, *p* = 0.13). Median seizure frequency fell from 41 to 5.1 seizures per month. There were two patients (25%) with a > 95% seizure reduction, and one patient with a slight increase in seizure frequency.

## 5. Discussion

In comparison to the results of the pivotal trial, our cohort experienced increased 1- and 2-year responder rates, a somewhat increased 1-year median percent reduction, and an increased 2-year median percent reduction (see Table 3). Our overall rates of seizure improvement, seizure freedom, and increase in seizures were similar (2, 4).

**Table 3:** Comparison of study cohort to previous trials.

	Comparison of study cohort to results of previous trial data^2,4^			Pivotal *N* = 191	Razavi et al. *N* = 150	Current cohort *N* = 30
1 year responder rate			43%	66%	70%
2 year responder rate				54%	77%	87%
1 year median percent reduction				44%	67%	57%
2 year median percent reduction				55%	75%	81%
Some improvement in seizures				82%		87%
Seizure freedom				9%	18%	7%
50% or greater increase in seizures				7%		10%

In comparing the two populations, age, sex, duration of epilepsy and rates of sEEG implantation were similar. Our population had fewer patients with prior epilepsy surgery, somewhat lower rates of multiple seizure foci, and an overall lower baseline seizure frequency. The finding in the pivotal trial that patients with increase in seizure frequency had a tendency to younger age (4) was not borne out in the present cohort.

In comparison to real-world data, our cohort had a lower proportion of patients with prior epilepsy surgery and higher rates of mesial temporal localization. Although only 57% of our patients underwent intracranial monitoring prior to implantation compared to 82% in the Razavi study (2), all our intracranial monitoring was performed with sEEG in contrast to a mix of sEEG and subdural grid and strip electrodes. Our cohort therefore is amongst the largest study of sEEG in RNS to date, where 73% of leads implanted were depth leads, compared with only 46% in the Razavi study.

Initial stim settings 200 Hz, 160 ms pulse width, 100 ms burst duration for charge density 0.5 uC are unchanged between the pivotal trial, the real-world data, and our cohort. Our subsequent programming was per provider discretion based on data from the original pivotal trial.

### 5.1. Subpopulation analysis

Subpopulation data was not reported in the pivotal trial except for mesial temporal onset. Subpopulation real-world data analysis found no difference in seizure frequency reduction depending on patient age, age at epilepsy onset, duration of epilepsy, location of seizure, brain MRI abnormalities, prior intracranial monitoring, prior epilepsy surgery, or prior VNS treatment.

Although our subpopulation analyses also largely did not reach significance, observational trends are relevant to guide future research. In evaluating the nine patients who were non-responders, 5 had undergone previous epilepsy surgery, 3 had prior VNS, and one had both. Age and epilepsy duration were similar between responders and non-responders. Additionally, all of the patients with baseline seizure frequency < 2/month were non-responders, including 3 with >700% increases in seizure frequency. This suggests there may be little room for improvement in this subgroup, and risk of worsening must be carefully considered.

Use of sEEG has a trend toward improved response rates, however this does not reach significance in either the total population or mesial temporal subpopulation analysis. The hypothesis that more accurate seizure localization prior to RNS implantation is based on the proposed therapeutic mechanism of RNS delivering stimulation to the site of ictal onset to disrupt seizure propagation. Assuming our observed trend of a 12% gain in response rate with sEEG compared to the whole cohort represents a true effect, we calculate a cohort size of 100 (50 with prior sEEG, 50 without) would be necessary to demonstrate statistical significance (α = 0.05, β = 0.1). Statistical calculations were made using G*Power 3 software (7).

Prior epilepsy surgery correlated with a poorer response rate in our study. This may indicate that continued disabling seizures after epilepsy surgery is a marker of refractory disease or kindling epilepsy networks beyond the identified and implanted ictal origin (8). Refractoriness may also be partially attributable to increasing years with epilepsy in this population, with an average of 24.1 (SD 7.6, range 10–34) years with epilepsy as compared to 20.5 years for the cohort as a whole. Inclusion of patients with low seizure frequency at baseline in this group may also contribute to poorer response rates. Notably, the majority of patients still experienced a reduction in seizure frequency. Further research is needed as to whether sEEG use may improve response rate.

Mesial temporal sclerosis may represent a slightly more refractory group; however this trend did not reach significance, nor did use of sEEG significantly affect response rate. History of prior VNS also has a trend towards lower response, suggesting this may also be a marker of more-refractory epilepsy. However, this trend did not reach significance, and presence of VNS is less likely to impact decisions regarding sEEG implantation.

### 5.2. Limitations

This is a small sample size limiting the ability to perform rigorous statistical analysis. Specifically, there is a risk to type I error with regards to poorer outcomes seen in the prior epilepsy surgery subpopulation analysis, and type II statistical error in the sEEG subpopulation analysis, as described above. We were unable to evaluate the confounding effects of continuous variables such as age and years with epilepsy on response rates in our subpopulation analysis, nor were we able to perform analysis of covariance. Our cohort was reasonably representative with respect to age, sex, and epilepsy localization but did not include any patients over the age of 55 nor patients with epilepsy duration <7 years, both of which factors might affect response rates. Our cohort also did not include specific epileptogenic lesions other than MTS and one patient with cortical dysplasia. Additionally, this as a single-center study may not be generalizable to all populations and centers.

Seizure frequency is tabulated by patient self-report, which may be unreliable especially in focal-unaware seizures. As a result, it is possible that some outliers reporting increased seizure frequency following implantation had a higher frequency at baseline which was inadequately captured without strict seizure-diary and RNS correlation. In patients with previous epilepsy surgery and/or VNS, data regarding preoperative seizure frequency and postoperative outcome are inconsistently available, as many patients were previously seen and treated by other centers. Finally, longitudinal data is less robust after 3 years post-implantation.

### 5.3. Conclusion

The present analysis of a single-center cohort supports the use of RNS in medically refractory epilepsy, with response rates matching or surpassing the original pivotal trial data and subsequent real-world study. Although the present study is limited by small study size, subpopulation analyses are encouraging for reasonable response rates in patients with previous sEEG vs. no sEEG and in mesial temporal vs. extratemporal pathology. It is possible that similar analysis with a larger sample size would reveal statistically significant differences in these groups that could influence management decisions. However, until larger studies are completed to confirm or refute the present findings, this study may be beneficial for other centers of similar volume in reconsidering the necessity of sEEG before RNS placement. Our study suggests additional research is needed to better evaluate the efficacy or limitations of RNS in patients with previous epilepsy surgery, and the relative utility of sEEG in this population. Finally, our study emphasizes the importance of internal review of surgical outcomes in order to better understand treatment failures and to identify trends that may inform future care in this complex population.

## Data availability statement

The original contributions presented in the study are included in the article/Supplementary material, further inquiries can be directed to the corresponding author.

## Ethics statement

Ethical approval was not required for the studies involving humans because it is a retrospective, observational study without identifiable subject information. The study was conducted in accordance with the local legislation and institutional requirements. Written informed consent for participation was not required from the participants or the participants’ legal guardians/next of kin in accordance with the national legislation and institutional requirements because retrospective, observational studies without identifiable subject information fall under exemption 45 CFR 46.104(d)(4) for human subject research regarding informed consent.

## Author contributions

MO contributed to analysis and interpretation of data and drafting the manuscript. MS contributed to the acquisition of data for the work, revising the draft critically for important intellectual content, and provided approval for publication of the content. TF contributed to conception and design of the study, completed the initial data acquisition, analysis, and interpretation, revised the draft critically for important intellectual content, provided final approval for publication of the content, and agrees to be accountable for all aspects of the work in ensuring that questions related to the accuracy or integrity of any part of the work are appropriately investigated and resolved. All authors contributed to the article and approved the submitted version.

## Conflict of interest

TF is on the speaker’s bureau for NeuroPace, Inc.

The remaining authors declare that the research was conducted in the absence of any commercial or financial relationships that could be construed as a potential conflict of interest.

## Publisher’s note

All claims expressed in this article are solely those of the authors and do not necessarily represent those of their affiliated organizations, or those of the publisher, the editors and the reviewers. Any product that may be evaluated in this article, or claim that may be made by its manufacturer, is not guaranteed or endorsed by the publisher.
