# Supplementary material for: Clinical outcomes following responsive neurostimulation implantation: a single center experience
Source: Front Neurol. 2023 Sep 22;14:1240380. doi: 10.3389/fneur.2023.1240380 (PMC10557254; doi:10.3389/fneur.2023.1240380)
Supplement: Supplementary file 1 [file Table_1.DOCX]

## Supplementary Table: Grouped Patient Characteristics

| Characteristic | All implanted (n=30) | No prior sEEG (n=13) | Prior sEEG (n=17) |
| --- | --- | --- | --- |
| Sex, % Female | 40 (12) | 46.2 (6) | 35.3 (6) |
| Age | 33.4 (10.7) 14-55 | 33.7 (10.9) 16-55 | 33.2 (10.6) 14-52 |
| Years with epilepsy | 20.5 (9.4) 7-41 | 21.3 (9.3) 8-41 | 19.9 (9.4) 7-36 |
| No. AEDs at time of implantation | 4.7 (1.6) 2-9 | 5.5 (1.5) 3-8 | 4.2 (1.5) 2-9 |
| Baseline seizures per month | 25.2 (29.8) 0.5-95 | 26.5 (32.6) 0.5-95 | 24.2 (27.5) 2.5-90 |
| Mesial temporal seizure onset | 66.7 (20) | 84.6 (11) | 52.9 (9) |
| Two seizure foci (vs one) | 43.3 (13) | 30.8 (4) | 52.9 (9) |
| Prior therapeutic surgery for epilepsy | 26.7 (8) | 46.2 (6) | 11.8 (2) |
| Prior VNS | 26.7 (8) | 23.1 (3) | 29.4 (5) |

Abbreviations: AED (antiepileptic drug), VNS (vagal nerve stimulator).

Data are mean (SD) minimum-maximum, or % (n).
